# Testing for the fitness benefits of natural transformation during community-embedded evolution

**DOI:** 10.1099/mic.0.001375

**Published:** 2023-08-01

**Authors:** Macaulay Winter, Klaus Harms, Pål Jarle Johnsen, Angus Buckling, Michiel Vos

**Affiliations:** ^1^​ European Centre for Environment and Human Health, University of Exeter Medical School, Environment and Sustainability Institute, Penryn Campus, TR10 9FE, UK; ^2^​ Microbial Pharmacology and Population Biology Research Group, Department of Pharmacy, Faculty of Health Sciences, UiT - The Arctic University of Norway, Tromsø, Norway; ^3^​ Department of Biosciences, University of Exeter, Penryn Campus, TR10 9FE, UK

**Keywords:** *Acinetobacter baylyi*, community evolution, evolution of sex, horizontal gene transfer, natural transformation, species interactions

## Abstract

Natural transformation is a process where bacteria actively take up DNA from the environment and recombine it into their genome or reconvert it into extra-chromosomal genetic elements. The evolutionary benefits of transformation are still under debate. One main explanation is that foreign allele and gene uptake facilitates natural selection by increasing genetic variation, analogous to meiotic sex. However, previous experimental evolution studies comparing fitness gains of evolved transforming- and isogenic non-transforming strains have yielded mixed support for the ‘sex hypothesis.’ Previous studies testing the sex hypothesis for natural transformation have largely ignored species interactions, which theory predicts provide conditions favourable to sex. To test for the adaptive benefits of bacterial transformation, the naturally transformable wild-type *Acinetobacter baylyi* and a transformation-deficient *∆comA* mutant were evolved for 5 weeks. To provide strong and potentially fluctuating selection, *A. baylyi* was embedded in a community of five other bacterial species. DNA from a pool of different *Acinetobacter* strains was provided as a substrate for transformation. No effect of transformation ability on the fitness of evolved populations was found, with fitness increasing non-significantly in most treatments. Populations showed fitness improvement in their respective environments, with no apparent costs of adaptation to competing species. Despite the absence of fitness effects of transformation, wild-type populations evolved variable transformation frequencies that were slightly greater than their ancestor which potentially could be caused by genetic drift.

## Introduction

Natural transformation is a process whereby bacteria actively take up free DNA from the environment during a physiological state termed competence, followed by the recombination of this DNA into the recipient’s genome (or its reconversion into extra-chromosomal genetic elements). Natural transformation has been demonstrated in 80+ species across divergent bacterial lineages [1] but is likely to be present in more species. Natural transformation can mediate the cell-to-cell transfer of large tracts of DNA, including virulence [2], antibiotic resistance [3, 4] and metabolic genes [5], making it one of the main prokaryote Horizontal Gene Transfer (HGT) mechanisms. The uptake of free DNA from the environment has been argued to provide three distinct (but not mutually exclusive) potential benefits to cells. First, as a source of nucleotides to be used for energy or building blocks (with recombination or maintenance of extrachromosomal DNA being a by-product) [6–8], second, to serve as templates for repairing genetic damage [6, 9–14], and third as a mechanism to create genetic variation [9, 15].

The genetic variation or ‘sex’ function of natural transformation has received most attention. Unlike many other HGT mechanisms, natural transformation is not mediated by mobile genetic elements but is solely under the control of the recipient cell [1, 16, 17] and therefore could be assumed to be adaptive. By recombining adaptive alleles and genes in the same genomic background, natural transformation can result in the avoidance of clonal interference, allowing populations to adapt more rapidly. Indeed, both mathematical modelling [18–20]; and laboratory evolution experiments [21–23]; have supported this hypothesis. However, there remains controversy about the sex function of transformation, and not all experimental studies have found that fitness of recombining wild-type cells increased after evolution compared to isogenic, non-recombinogenic mutants. For instance, evolution experiments utilising the model system *Acinetobacter baylyi* found transformation-mediated fitness benefits either to be present [24], equivocal [25, 26] or absent [27, 28]. Multiple studies found the ability to transform was lost during experimental evolution, indicating that any potential recombination-mediated fitness benefits were outweighed by the cost of maintaining the molecular machinery involved [25, 27].

Studies to date lack interactions with multiple competitors that almost certainly characterise most natural situations. This could be an important shortcoming, as for sex to remain selectively advantageous it is necessary for selection to be strong and dynamic [29, 30], and interspecific competitors could greatly influence both these requirements [31, 32]. For example, if species evolve niche divergence, then the strength of interaction will decline over time (in contrast to host-parasite coevolution, where strength is maintained) [33]. However, while interspecific competition can create fluctuating conditions, it can also constrain evolution [34]. Experimental evolution approaches have hitherto relied on evolving recombining clones in isolation. To study the evolutionary benefits of natural transformation in the context of species interactions, we here experimentally evolve a transformable *A. baylyi* wild-type and a non-transformable isogenic *∆comA* mutant for 5 weeks in the presence of other species (i.e*.* under biotic conditions) or alone (i.e*.* under abiotic conditions). The *comA* gene encodes the transmembrane protein comA which facilitates transport of ssDNA through the inner membrane into the cytoplasm [35]. Deletion of *comA* reduces transformation frequency in *A. baylyi* below the detectable limit [35]. Specifically, we use a system of five bacterial species which have been previously shown to stably coexist [36–38]. Our experimental evolution approach allows us to test 1) whether the evolved wild-type strain will be fitter than its non-recombining counterpart, specifically after evolution under biotic conditions and 2) whether transformation rate of the evolved wild-type has changed in response to these treatments.

## Methods

### *Acinetobacter baylyi* ADP1 constructs

Two variants of the wild-type *A. baylyi* ADP1 strain with chromosomally encoded GFP and RFP, respectively were constructed using fluorescence::AMR cassettes derived from strains gifted by the Charpentier lab (Claude Bernard University, Lyon), and Hasty lab (University of San Diego, California), respectively. Briefly, the *sfGFP::apraR* cassette (Charpentier and Laaberki, unpublished) and *mCherry::specR* [39] cassettes were amplified via PCR with 1 kb flanking regions homologous to the *att*Tn*7* locus and a putative prophage p4 region, respectively. Primers used for the *sfGFP::apraR* and *mCherry::specR* cassettes were 5′-AAAGCCAATCGCTGACAGATGGTGG-3′, 5′-TTGGTCAGTGCCTGTCTTGCTGGTGAGCCGGTACGC-3′, and 5′-TCACCTGCATCCACTCAAGTGTCGTTT-3′, 5′-AAAGCCAATCGCTGACAGATGGTGG-3′, respectively (Integrated DNA Technologies, USA). PCR products were added to *A. baylyi* in LB Miller broth (Formedium, England) at 37 °C and 180 r.p.m. for 24 h at 1 µg ml^−1^ to allow for chromosomal recombination of PCR amplificates via natural transformation. Transformants were isolated by plating on LB agar containing 240 µg ml^−1^ apramycin (Duchefa, The Netherlands), or 360 µg ml^−1^ spectinomycin (Melford, UK), respectively. Next, non-competent counterparts of each fluorescent strain were generated by deletion of the *comA* gene via sequential natural transformation with linearised plasmids pKHNH6 and pKHNH3. Plasmids pKHNH6 and pKHNH3 were linearised prior to transformation using restriction enzymes EcoRV (Promega, USA) and KpnI (Fisher, USA), respectively. pKHNH6 carried a *comA* ^+^::(*nptII sacB*) allele embedded in its natural flanking regions, and natural transformation of the respective fluorescence-marked *A. baylyi* strains by linearized plasmid DNA resulted in transformation-proficient isolates that were kanamycin-resistant and sensitive to 50 g l^−1^ sucrose. Transformation of those respective isolates by pKHNH3 (carrying a *∆comA* allele) resulted in a sucrose-resistant, kanamycin-susceptible and transformation-deficient strains, respectively. Deletion of *comA* was verified using agar containing 50 g l^−1^ sucrose, agar containing 10 ug ml^−1^ kanamycin, and by PCR using primers 5′-TTGGTGTGATTGGTACGGTGGCTGGTGC-3′ and 5′-CTTGCAGACGATTGCTTACCTCAGCACTCGG-3′. The non-competent *A. baylyi* strains were confirmed to be non-transformable at the detectable limit (10^−7^) in all (six) technical replicates.

### Five-species community

The five-species community was composed of compost isolates belonging to the genera *Pseudomonas, Achromobacter, Variovorax, Ochrobactrum* and *Stenotrophomonas* (as identified by 16S rRNA sequencing) [36, 38]; (Table 1).

**Table 1.** List of strains used in this study

*Strain*	*Fluorescence label*	*Naturally competent?*	*Antibiotic resistance marker*	*Used as*
*wild-type A. baylyi ADP1*	sfGFP (green)	Yes	Apramycin (480 ug ml^−1^)	Focal strain in evolution experiment
*∆comA A. baylyi ADP1*	sfGFP (green)	No	Apramycin (480 ug ml^−1^)	Focal strain in evolution experiment
*∆comA A. baylyi ADP1*	mCherry (red)	No	Spectinomycin (600 ug ml^−1^)	Common competitor for fitness assays
* Achromobacter *	None	No	n/a	Community member
* Ochrobactrum *	None	No	n/a	Community member
* Pseudomonas *	None	No	n/a	Community member
* Stenotrophomonas *	None	No	n/a	Community member
* Variovorax *	None	No	n/a	Community member
*A. sp. 01B0, KmR isolate 2*.	None	No	n/a	DNA Donor
*A. sp. 26B2, isol. 3*.	None	Yes	n/a	DNA Donor
*A. sp. 423D, isol. 3*.	None	Yes	n/a	DNA Donor
*A. sp. 48A1, isol. 3*.	None	No	n/a	DNA Donor
*A. sp. 511B, isol. 5*.	None	No	n/a	DNA Donor
*A. sp. 56A1, isol. 3*.	None	No	n/a	DNA Donor
*A. sp. 62A1, isol. 3*.	None	Yes	n/a	DNA Donor
*A. sp. 63A1, isol. 7*.	None	Yes	n/a	DNA Donor
*A. sp. 66A1, isol. 1*.	None	No	n/a	DNA Donor
*A. sp. 71A1, isol. 4*.	None	No	n/a	DNA Donor
*A. sp. 81A1, isol. 2*.	None	No	n/a	DNA Donor
*A. sp. 85A1, isol.3*.	None	Yes	n/a	DNA Donor
*A. sp. A06, isol. 7*.	None	Yes	n/a	DNA Donor
*A. sp. A3-6, isol. 3*.	None	No	n/a	DNA Donor
*A. sp. AD512A, isol. 4*.	None	No	n/a	DNA Donor
*A. baumannii AZR3410, isol. 1*.	None	No	n/a	DNA Donor
*A. sp. AZR54, isol. 8*.	None	Yes	n/a	DNA Donor
*A. calcoaceticus AZR583, isol. 9*.	None	Yes	n/a	DNA Donor
*A. sp. P1-6, isol. 3*.	None	Yes	n/a	DNA Donor
*A. baumannii clinical isolate FZ21*	None	Unknown	n/a	DNA Donor

### Evolution experiment

**Fig. 1.**
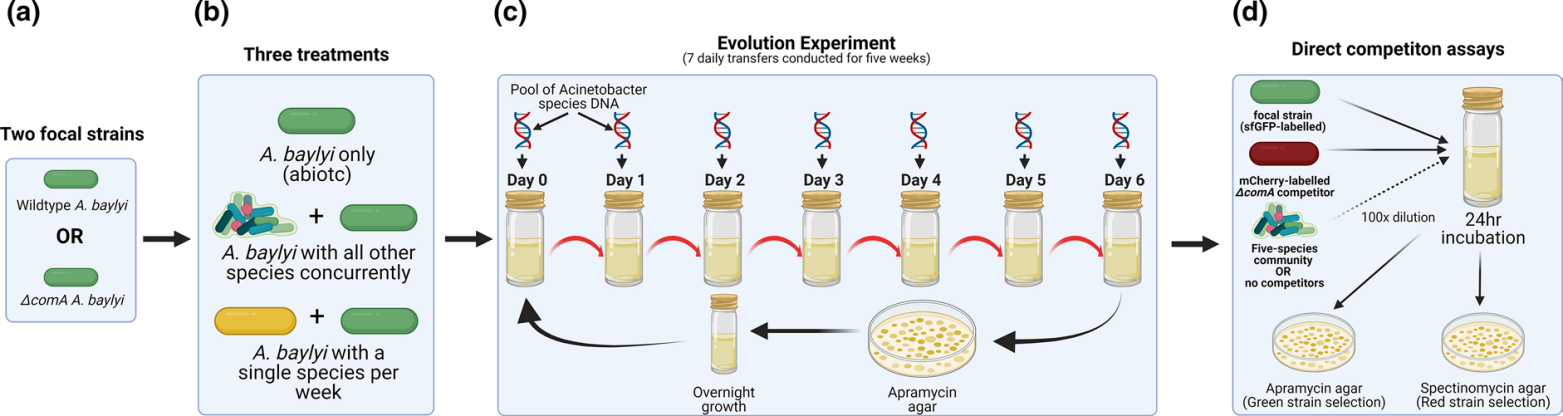
Illustration of the evolution experiment and direct competition assays. (a) Two focal strains (competent wild-type and isogenic non-competent counterpart) were cultured separately in their respective treatment conditions. (**b**) Focal strains were subjected to two treatment conditions: monoculture (abiotic) or co-culture with five competitor species. (c) Cultures were propagated by replacing spent broth with fresh media resulting in an approximately 34-fold dilution each passage. After the sixth passage, all species except the focal strain are killed off using LB agar amended with apramycin. The focal strain and freezer stocks of the competitors were allowed to grow to maximum density in LB broth before being inoculated together for another week of passaging. (d) After five full weeks of passaging, the focal strains are selected for again with use of apramycin agar and frozen in 25 % glycerol at −70 °C until tested against a common competitor to measure relative fitness. Figure created with BioRender.com.

The apramycin resistant wild-type and *∆comA A. baylyi* ADP1 focal strains were separately propagated in three distinct competition environments with sixfold replication. Experimental treatments included competition against none, or all five of the five-species community concurrently (Fig. 1). Cultures were grown in 25 % TSB medium at 28 °C in static conditions in glass microcosms with one layer of ColiRoller glass beads (Millipore, Merck, USA) covering the base of the microcosm to provide additional spatial structure. Instead of transferring a small volume to new microcosms, spent nutrient broth was replaced with fresh broth in the same microcosms, resulting in approximately 34-fold daily dilutions. This approach maintained spatial structure and saved on glass and plastic waste [40]. Every seventh day, cultures were vortexed, diluted ten-fold and plated on LB agar plates containing 240 µg ml^−1^ apramycin to select for the green-fluorescent wild-type or *∆comA A. baylyi* focal strain. After 24 h of incubation at 37 °C, the bacterial lawn from each individual replicate was scraped off with a sterile loop and transferred to a new microcosm for overnight growth (reaching stationary phase). Overnight cultures of the five competitor species were also made in LB broth at this time from −70 °C freezer stocks. Equal volumes of overnight cultures of the focal *A. baylyi* strain and competitor species (where appropriate; Table 1) totalling 100 µl was added to a new microcosm containing 9.9 ml 25 % TSB, commencing the next week of transfers. DNA as a substrate for transformation sourced from a pool of 20 *Acinetobacter* strains was added at the point of nutrient replenishment each day for each treatment (see next section). At the end of the final week of transfers, cultures were plated on LB agar supplemented with 240 µg ml^−1^ apramycin where 100 colonies were picked and pooled for overnight growth and later frozen at −70 °C in 25 % glycerol prior to use in subsequent competition and transformation assays (Fig. 1).

### Donor DNA

Twenty *Acinetobacter* strains (Table 1; [41, 42]) were cultured separately in LB broth overnight. Cultures were then pooled in equal volumes and lysed following the Qiagen Genomic DNA Handbook (April 2012) protocol. Combined DNA from the eluate was precipitated by adding two volumes of ice-cold ethanol and centrifuged at 26 000 ***g*** for 15 min to pellet the DNA. DNA was dissolved in TE buffer to a final concentration of 227.2 ng µl^−1^ (Nanodrop 2000, Thermo Scientific) by heating at 50 °C and stirring for 16 h. DNA was frozen at −20 °C in single-use aliquots for addition to each daily transfer. When used during the evolution period, DNA was diluted to a final concentration of 250 ng ml^−1^ (the saturating concentration of genomic DNA for transformation in *A. baylyi* [43]). The raw and annotated DNA sequencing data for the 19 strains were deposited at the European Nucleotide Archive under BioProject accession number PRJEB55833 [44].

### Competition assays

For all replicates of the five concurrent species community treatment and the abiotic treatment, 100 evolved green-fluorescent wild-type and *∆comA A. baylyi* clones were picked, pooled and frozen at –80 °C before use. A mixture containing equal volumes of the green-fluorescent *A. baylyi* pool, a *∆comA* red-fluorescent *A. baylyi *, and members of the five species community (where appropriate) was produced for each replicate. One hundred microlitres of mixed culture was immediately inoculated to 9.9 ml of 25 % TSB and grown for 24 h in glass microcosms at 28 °C with no agitation. Each of the six replicate evolved populations per treatment were competed with the differentially marked *∆comA* strain in conditions identical to treatments in the evolution experiment (i.e. containing either all or none of the five competitor species). Samples were plated 0 h and 24 h after inoculation on LB agar supplemented with either apramycin (240 µg ml^−1^) or spectinomycin (360 µg ml^−1^) to determine the densities of the evolved (green)- and *∆comA* (red)-fluorescent *A. baylyi *, respectively (Fig. 1).

### Transformation assays

Red fluorescent, spectinomycin-resistance conferring marker DNA was obtained by heating overnight cultures of the *A. baylyi* ADP1 *mCherry::specR* at 70 °C for 1.5 h and centrifuging at 2500 ***g*** for 15 min and resuspending in reduced volume to produce a 50× concentration of lysate. Lysate was stored at −20 °C for up to 1 month before downstream application. Freezer stocks of 100 pooled clones for each endpoint population were inoculated in LB broth and grown overnight at 37 °C. Cultures were diluted ten-fold with LB broth supplemented with cell lysate at a final concentration of 2.5× maximum cell density and incubated for 3 h at 37 °C and 180 r.p.m. Cells were then plated on LB agar supplemented with 240 µg ml^−1^ apramycin and 360 µg ml^−1^ spectinomycin, and non-selective LB agar and incubated for 48 h at 28 °C. Transformation frequency was calculated by dividing the c.f.u. ml^−1^ of the transformed (doubly fluorescent and dually-AMR) population by the total population c.f.u. ml^−1^. As a control for spontaneous spectinomycin resistance mutations, we included a treatment where no *mCherry::specR* DNA was added.

### Statistical analysis

Normal distribution of the residuals for data used in statistical modelling was verified using Shapiro-Wilks tests. To determine the relative fitness of evolved lines relative to the unevolved control in competition experiments, the selection-rate constant was calculated as described in [45]. Selection-rate constant estimates were analysed with emmeans tests using a linear model as input data (treatments tested within assay conditions and grouped by genotype and evolution treatment conditions). Because relative fitness values were often negative (i.e. the common competitor displayed greater fitness than the evolved focal strains), analyses in this assay were conducted using the selection-rate constant in lieu of the relative fitness parameter [45]. As ancestral populations were not significantly different to each other in fitness as a function of genotype (paired t-tests; biotic assay conditions, *P*=0.35, abiotic assay conditions, *P*=0.74), fitness measurements of the evolved populations were standardised to ancestors by subtracting the ancestral selection-rate constant (averaged for both genotypes) from that of the evolved population in all analyses. Standardised selection rates significantly different to zero demonstrate fitness change.

Generalised statements about treatment effects observed in fitness assays were made using linear models. Model selection was achieved using backward stepwise regression. Model residuals were checked using the DHARMa package v0.4.5 [46]. Emmeans testing was conducted using the emmeans package v1.8.4–1 [47]. All biological replicates for the selection-rate constant analyses and transformation frequency measurements were measured in triplicate and averaged before downstream analyses. Transformation frequencies were analysed non-parametrically with Wilcoxon tests as the data were not normally distributed (Shapiro-Wilks tests, *P*<0.001). The mean transformation frequency of the wild-type ancestor was subtracted from the mean transformation frequencies of all samples and the resulting values were tested for significant differences to zero. T-tests, Wilcoxon, Kruskal-Wallis and Levene testing was conducted using the rstatix package v0.7.0 [48]. In all analyses, a *p* value of less than 0.05 was considered significant. Multiple testing was corrected for with false discovery rate (FDR) correction.

## Results

### Natural transformation does not provide fitness benefits in a community context

**Fig. 2.**
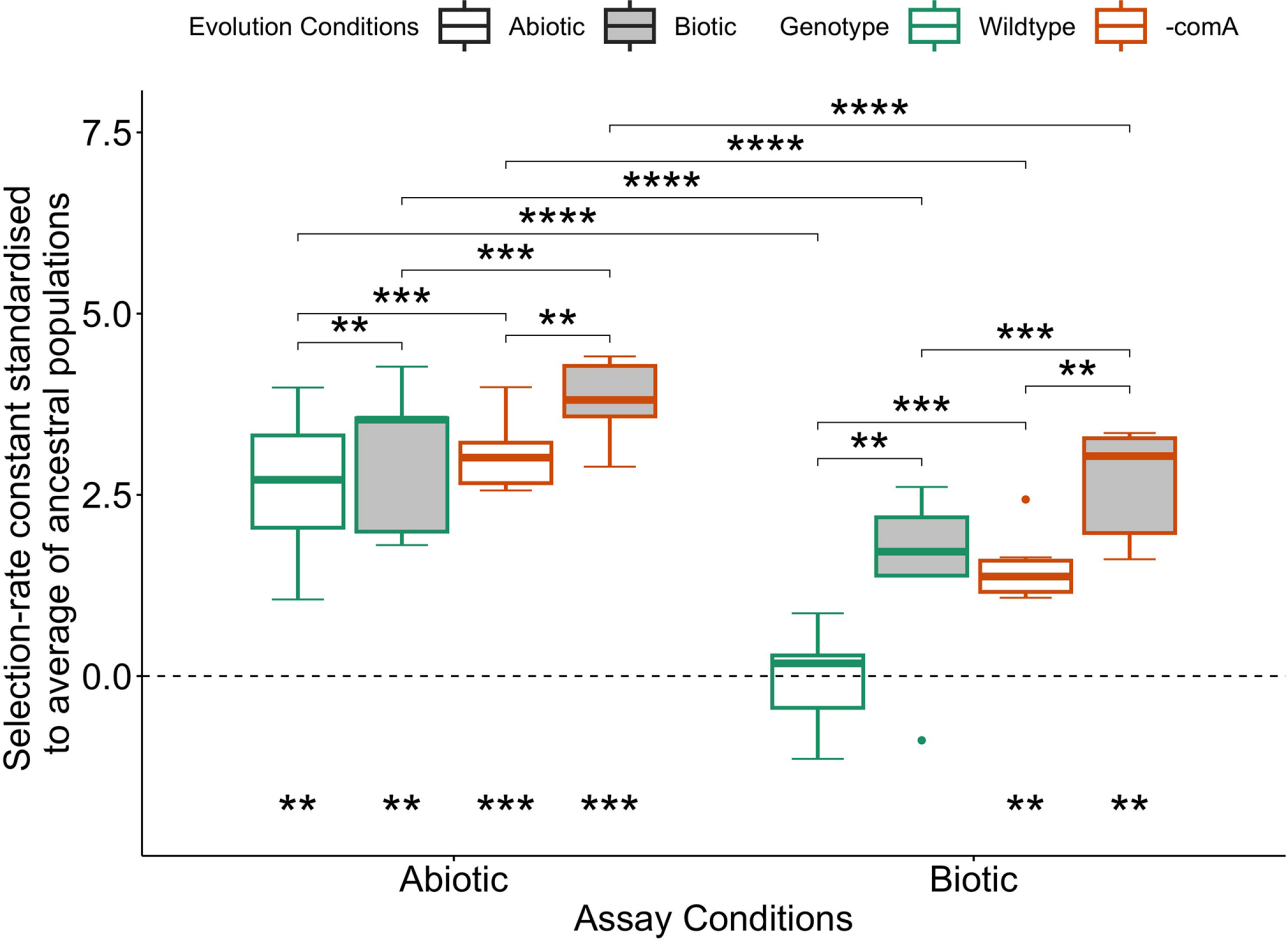
Selection-rate constants of evolved populations standardised to ancestral populations in respective assay conditions (y=0). Asterisks describe significant differences (emmeans test) between evolved populations within the same assay conditions. Asterisks (bottom) describe significant differences (t-test) between evolved populations and zero. Top, middle, and bottom bands of the boxes denote the 75 % quartile, the median, and the 25 % quartile, respectively. Box whiskers are 1.5× the interquartile range. Fitness differences of each of the six biological replicates per treatment were measured in triplicate. (*, *P*<0.05; **, *P*<0.01; ***, *P*<0.001).

To test whether natural transformation favours adaptation in a biotic community context compared to an abiotic environment, we evolved a recombining wild-type *A. baylyi* and an isogenic non-recombining *∆comA* mutant supplemented with DNA extracted from a pool of conspecifics as a substrate for natural transformation. This five species community has been shown to stably coexist in 1/64 strength tryptic soy broth (TSB), and 28 °C with weekly passages [38], but was found to also exist with *A. baylyi* stably in 25 % TSB medium with daily transfers (results not shown). After 5 weeks of evolution (resulting in approximately 180 generations of log phase growth), changes in fitness of each evolved line relative to a single unevolved, differentially marked *∆comA* strain were measured using pairwise competition assays. Populations were assayed in both presence and absence of competitors. All populations increased in fitness relative to ancestral populations (one sample t-tests: *P*<0.01, corrected for multiple testing, Fig. 2, Table 2), except for the wild-type strains evolved in abiotic and biotic conditions when assayed in biotic conditions (*P*=0.486, and *P*=0.058, respectively; Fig. 2, Table 2).

**Table 2.** Emmeans of standardised selection-rate constants for assay conditions, genotypes, and evolution conditions. Degrees of freedom were calculated using the Kenwood-Roger method. A 0.95 confidence interval was used for all samples

*Evolution conditions*	*Assay conditions*	*Genotype*	*emmean*	*SE*	*df*	*lower.CL*	*upper.CL*
*Abiotic*	Abiotic	Wild-type	2.17	0.254	42	1.654	2.681
*Biotic*	Abiotic	Wild-type	3.11	0.266	42	2.569	3.643
*Abiotic*	Biotic	Wild-type	0.41	0.254	42	−0.104	0.923
*Biotic*	Biotic	Wild-type	1.35	0.266	42	0.811	1.885
*Abiotic*	Abiotic	-comA	3.17	0.254	42	2.658	3.685
*Biotic*	Abiotic	-comA	4.11	0.254	42	3.597	4.624
*Abiotic*	Biotic	-comA	1.41	0.254	42	0.901	1.928
*Biotic*	Biotic	-comA	2.35	0.254	42	1.839	2.866

Selection of the most parsimonious linear model was achieved by backward stepwise regression. Linear models revealed no interactions between explanatory variables genotype, evolutionary conditions, and assay conditions when predicting fitness increases (*P*>0.05). Genotype, assay conditions, and evolution conditions are significant explanatory variables for model predictions (F=15.215, df=1, *P*<0.001; F=46.751, df=1, *P*<0.0001; and F=13.277, df=1, *P*<0.001, respectively). All evolved population groups demonstrated greater adaptation to the abiotic environment than the biotic environment irrespective of evolution conditions (emmeans pairwise testing, *P*<0.0001; Fig. 2, Table 3). Both wild-type and *∆comA* strains evolved in the biotic environment are better adapted to both test environments than their counterparts evolved in the abiotic environment (emmeans pairwise testing, *P*<0.001; Fig. 2; Table 3). The evolved *∆comA* populations are better adapted to the experimental environments relative to the evolved wild-type when assayed in either environment (emmeans pairwise testing, *P*<0.001; Fig. 2, Table 3). This observation applies to evolution in either environment.

**Table 3.** Pairwise comparisons of evolved populations’ relative fitness gains after 5 weeks’ evolution in biotic or abiotic conditons (emmeans test). Evolved populations were tested for fitness gains in biotic and abiotic conditions

*Evolution Condition*	*Assay Condition*	*Genotype*		*Evolution Condition*	*Assay Condition*	*Genotype*	*Estimate*	*SE*	*df*	*t.ratio*	*p.value*
*Biotic*	Abiotic	Wild-type	–	Abiotic	Abiotic	-comA	−0.0662	0.372	42	−0.178	0.8599
*Biotic*	Biotic	Wild-type	–	Abiotic	Biotic	-comA	−0.0662	0.372	42	−0.178	0.8599
*Abiotic*	Abiotic	Wild-type	–	Biotic	Biotic	-comA	−0.1856	0.439	42	−0.423	0.7266
*Abiotic*	Abiotic	Wild-type	–	Abiotic	Biotic	-comA	0.7529	0.364	42	2.069	0.0501
*Biotic*	Abiotic	Wild-type	–	Biotic	Biotic	-comA	0.7529	0.364	42	2.069	0.0501
*Abiotic*	Abiotic	Wild-type	–	Biotic	Biotic	Wild-type	0.8191	0.364	42	2.251	0.0361
*Abiotic*	Abiotic	-comA	–	Biotic	Biotic	-comA	0.8191	0.364	42	2.251	0.0361
*Abiotic*	Abiotic	Wild-type	–	Biotic	Abiotic	Wild-type	−0.9385	0.258	42	−3.644	0.001
*Abiotic*	Biotic	Wild-type	–	Biotic	Biotic	Wild-type	−0.9385	0.258	42	−3.644	0.001
*Abiotic*	Abiotic	-comA	–	Biotic	Abiotic	-comA	−0.9385	0.258	42	−3.644	0.001
*Abiotic*	Biotic	-comA	–	Biotic	Biotic	-comA	−0.9385	0.258	42	−3.644	0.001
*Biotic*	Abiotic	Wild-type	–	Abiotic	Biotic	-comA	1.6914	0.453	42	3.738	0.0009
*Abiotic*	Abiotic	Wild-type	–	Abiotic	Abiotic	-comA	−1.0046	0.258	42	−3.901	0.0006
*Biotic*	Abiotic	Wild-type	–	Biotic	Abiotic	-comA	−1.0046	0.258	42	−3.901	0.0006
*Abiotic*	Biotic	Wild-type	–	Abiotic	Biotic	-comA	−1.0046	0.258	42	−3.901	0.0006
*Biotic*	Biotic	Wild-type	–	Biotic	Biotic	-comA	−1.0046	0.258	42	−3.901	0.0006
*Biotic*	Biotic	Wild-type	–	Abiotic	Abiotic	-comA	−1.8237	0.453	42	−4.03	0.0005
*Abiotic*	Abiotic	Wild-type	–	Abiotic	Biotic	Wild-type	1.7575	0.257	42	6.837	<0.0001
*Abiotic*	Abiotic	Wild-type	–	Biotic	Abiotic	-comA	−1.9431	0.356	42	−5.46	<0.0001
*Biotic*	Abiotic	Wild-type	–	Abiotic	Biotic	Wild-type	2.696	0.364	42	7.409	<0.0001
*Biotic*	Abiotic	Wild-type	–	Biotic	Biotic	Wild-type	1.7575	0.257	42	6.837	<0.0001
*Abiotic*	Biotic	Wild-type	–	Abiotic	Abiotic	-comA	−2.7621	0.364	42	−7.591	<0.0001
*Abiotic*	Biotic	Wild-type	–	Biotic	Abiotic	-comA	−3.7006	0.439	42	−8.43	<0.0001
*Abiotic*	Biotic	Wild-type	–	Biotic	Biotic	-comA	−1.9431	0.356	42	−5.46	<0.0001
*Biotic*	Biotic	Wild-type	–	Biotic	Abiotic	-comA	−2.7621	0.364	42	−7.591	<0.0001
*Abiotic*	Abiotic	-comA	–	Abiotic	Biotic	-comA	1.7575	0.257	42	6.837	<0.0001
*Biotic*	Abiotic	-comA	–	Abiotic	Biotic	-comA	2.696	0.364	42	7.409	<0.0001
*Biotic*	Abiotic	-comA	–	Biotic	Biotic	-comA	1.7575	0.257	42	6.837	<0.0001

### Transformation frequency did not decrease after evolution

**Fig. 3.**
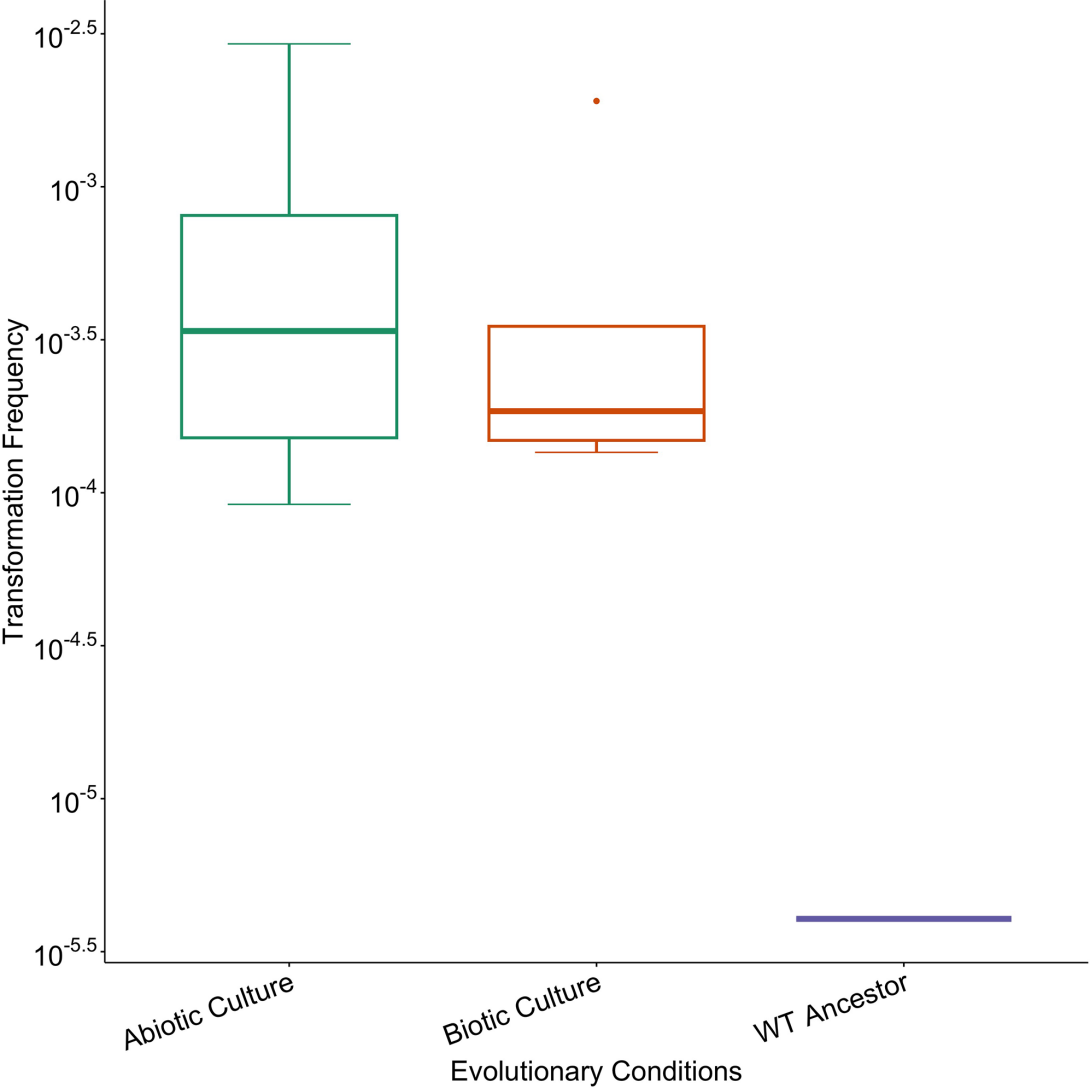
Treatment-level transformation frequencies of ancestral and evolved wild-type populations using heat-killed cell lysate. Transformation frequencies were measured in triplicate per biological replicate. The Abiotic Culture, Biotic Culture, and WT ancestor treatments had 6, 5, and one biological replicates, respectively.

To test whether the lack of fitness difference of the wild-type strain stemmed from a possible loss of natural transformation, we conducted transformation assays for the abiotic treatment and the five species community treatment (Fig. 1). No significant differences in transformation frequency were found between the evolved wild-type lineages across the two evolution environments or the ancestor (Wilcoxon one sample test, *P*>0.05; Fig. 3). While transformation frequency did not change significantly, observed transformation frequencies were higher in all evolved wild-type populations than in the ancestor.

## Discussion

Here we tested if recombination mediated by natural transformation in *A. baylyi* provided adaptive benefits under biotic and abiotic experimental conditions. All replicate populations in either treatment increased fitness compared to their ancestors when assayed in abiotic conditions (Fig. 2). Populations of the non-recombining (*∆comA*) genotype also show increased fitness relative to their ancestor when assayed under biotic conditions regardless of evolutionary conditions. This contrasts with the recombining wild-type, which does not show significant increases in fitness after evolution in the biotic environment, contrary to our hypothesis that sex is advantageous in the presence of other species. However, the absence of significant fitness increases observed in the wild-type which was evolved and tested in biotic conditions are probably due to a single tested population which is less fit than the ancestor, lowering the estimation of the mean (Fig. 2). Further, no significant differences in fitness between evolved lineages were found when comparing across genotypes and assaying in biotic conditions. Therefore, this study has not found a clear and significant beneficial (or deleterious) effect of natural transformation on adaptative evolution.

There is clear trade-off asymmetry displayed by evolving the wild-type *A. baylyi* in the presence or absence of the five competitor species (biotic and abiotic conditions, respectively). Evolution of the wild-type under biotic conditions did not constrain adaptation to an abiotic environment, but the converse is not true (Fig. 2). This is because the factors in abiotic conditions (growth media, temperature, oxygen availability, spatial structure) were also consistent in the biotic condition and are therefore selected for in the biotic environment. However, there may be some adaptive mutations that arise under biotic conditions which are not advantageous in the abiotic environment, and therefore were not selected for during evolution in abiotic conditions [49]. Our data show that evolution in a biotic environment best prepares a population for a biotic environment, and does not constrain adaptation to an abiotic environment, regardless of the population’s ability to naturally transform.

**Fig. 4.**
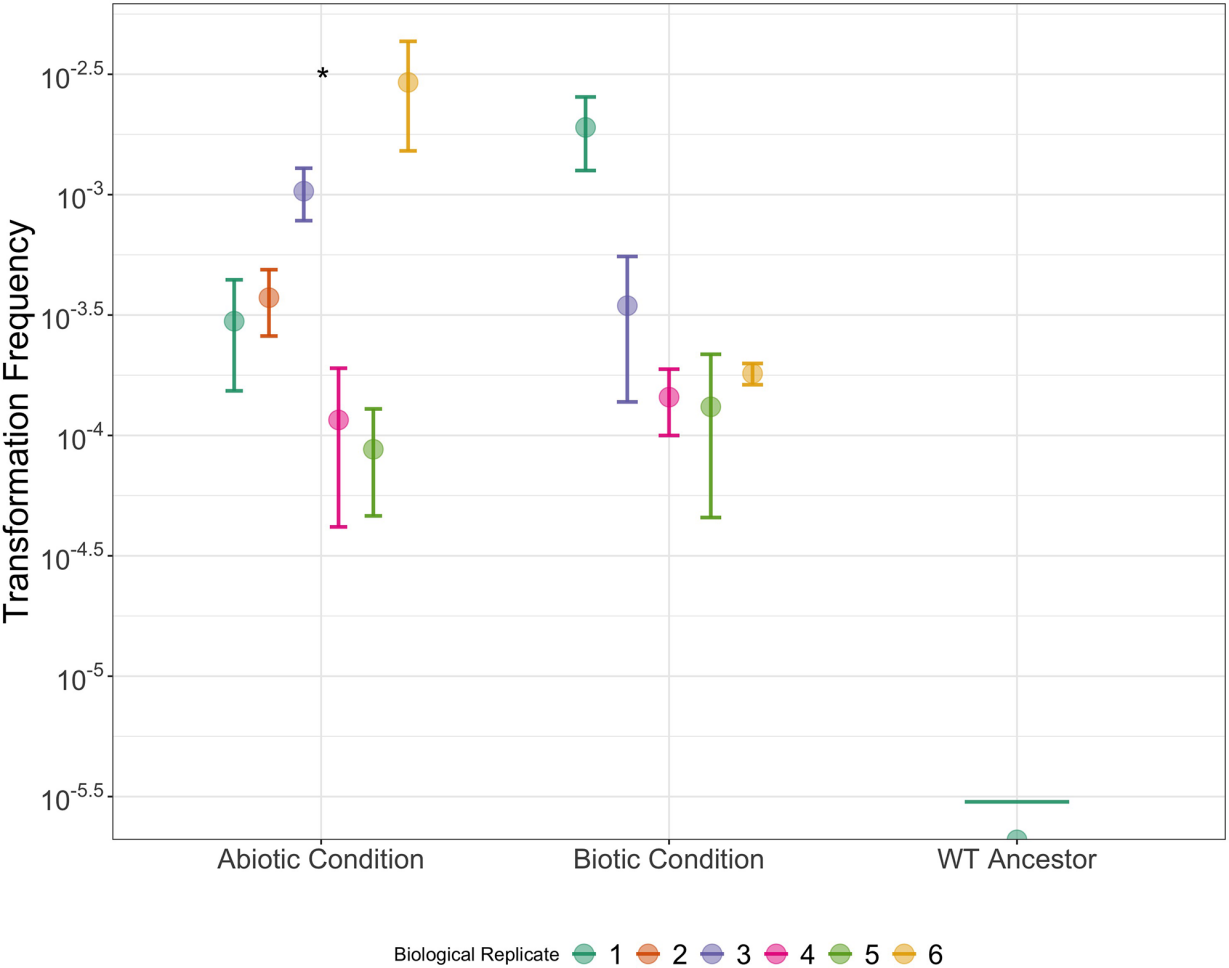
Variation of transformation frequencies of biological replicates within evolutionary treatment groups for heat-killed cell lysate. Transformation frequencies were measured in triplicate per biological replicate. Points are biological replicates and bars are means±one standard error. Asterisks denote significantly different transformation frequencies between intra-treatment biological replicates (Kruskal-Wallis, *P*<0.05).

Interestingly, mean transformation frequencies of all tested populations increased relative to the transformable ancestor, although this was not statistically significant. This contrasts with previous studies where transformation frequencies declined markedly after experimental evolution [26, 27]. The maintenance, and potential increase of transformability in all evolved populations is not consistent with a selective benefit. As the ability to transform was not selected against either, it is possible it may have increased because of genetic drift. The increase of transformation frequency at varying rates per population (Fig. 4) suggests that significant differences both within and between treatment groups may occur after a more prolonged evolution period.

Taken together, these findings show no significant evolutionary benefits of transformation in *A. baylyi *. This does not mean that no bacterial species use transformation to facilitate faster adaptation, as the physiological contexts and molecular mechanisms of natural transformation vary across species [1]. Specifically, there is convincing evidence supporting the sex hypothesis for transformation in *H. pylori* [21–23]. There is no doubt that transformation can give an immediate adaptive benefit to *A. baylyi* such as in the contexts of antimicrobial resistance acquisition [6, 24, 50, 51], but its long-term benefits remain uncertain. The selection exerted by other species in this study may not have been sufficiently strong, or benefits derived from natural transformation might have accrued only at the start of the experiment before fitness was assayed. It is possible that future experiments utilising different growth conditions, interactions with live DNA donors, and reduced doubling rates of cells (limiting the frequency and contribution of mutation for adaptation), could still demonstrate the adaptive benefit of natural transformation in *A. baylyi *.
